# Supplementary material for: The determinants and engagement patterns of chaperones and chauffeurs by Australian doctors in after-hours house-call services
Source: PeerJ. 2017 Apr 26;5:e3218. doi: 10.7717/peerj.3218 (PMC5408717; doi:10.7717/peerj.3218)
Supplement: Supplemental Information 1 [file peerj-05-3218-s001.docx]

1. **CROSS TABS DRIVING HABITS AND LOCATION**

**chauffeur driven only * Adelaide recoded**

|  | | | | | |
| --- | --- | --- | --- | --- | --- |
|  | | | Adelaide recoded | | Total |
|  |  |  | Yes | No |  |
| chauffeur driven only | yes | Count | 17 | 57 | 74 |
|  |  | Expected Count | 23.1 | 50.9 | 74.0 |
|  | no | Count | 23 | 31 | 54 |
|  |  | Expected Count | 16.9 | 37.1 | 54.0 |
| Total | | Count | 40 | 88 | 128 |
|  |  | Expected Count | 40.0 | 88.0 | 128.0 |

| **Chi-Square Tests** | | | | | |
| --- | --- | --- | --- | --- | --- |
|  | Value | df | Asymptotic Significance (2-sided) | Exact Sig. (2-sided) | Exact Sig. (1-sided) |
| Pearson Chi-Square | 5.593^a^ | 1 | .018 |  |  |
| Continuity Correction^b^ | 4.717 | 1 | .030 |  |  |
| Likelihood Ratio | 5.563 | 1 | .018 |  |  |
| Fisher's Exact Test |  |  |  | .021 | .015 |
| Linear-by-Linear Association | 5.550 | 1 | .018 |  |  |
| N of Valid Cases | 128 |  |  |  |  |
| a. 0 cells (0.0%) have expected count less than 5. The minimum expected count is 16.88. | | | | | |
| b. Computed only for a 2x2 table | | | | | |

**chauffeur driven only * Brisbane Area Recoded**

|  | | | Brisbane Area Recoded | | Total |
| --- | --- | --- | --- | --- | --- |
|  |  |  | Yes | No |  |
| chauffeur driven only | yes | Count | 31 | 43 | 74 |
|  |  | Expected Count | 20.2 | 53.8 | 74.0 |
|  | no | Count | 4 | 50 | 54 |
|  |  | Expected Count | 14.8 | 39.2 | 54.0 |
| Total | | Count | 35 | 93 | 128 |
|  |  | Expected Count | 35.0 | 93.0 | 128.0 |

| **Chi-Square Tests** | | | | | |
| --- | --- | --- | --- | --- | --- |
|  | Value | df | Asymptotic Significance (2-sided) | Exact Sig. (2-sided) | Exact Sig. (1-sided) |
| Pearson Chi-Square | 18.687^a^ | 1 | .000 |  |  |
| Continuity Correction^b^ | 16.991 | 1 | .000 |  |  |
| Likelihood Ratio | 21.033 | 1 | .000 |  |  |
| Fisher's Exact Test |  |  |  | .000 | .000 |
| Linear-by-Linear Association | 18.541 | 1 | .000 |  |  |
| N of Valid Cases | 128 |  |  |  |  |
| a. 0 cells (0.0%) have expected count less than 5. The minimum expected count is 14.77. | | | | | |
| b. Computed only for a 2x2 table | | | | | |

**chauffeur driven only * Gold Coast recoded**

|  | | | Gold Coast recoded | | Total |
| --- | --- | --- | --- | --- | --- |
|  |  |  | Yes | No |  |
| chauffeur driven only | yes | Count | 1 | 73 | 74 |
|  |  | Expected Count | 9.3 | 64.8 | 74.0 |
|  | no | Count | 15 | 39 | 54 |
|  |  | Expected Count | 6.8 | 47.3 | 54.0 |
| Total | | Count | 16 | 112 | 128 |
|  |  | Expected Count | 16.0 | 112.0 | 128.0 |

| **Chi-Square Tests** | | | | | |
| --- | --- | --- | --- | --- | --- |
|  | Value | df | Asymptotic Significance (2-sided) | Exact Sig. (2-sided) | Exact Sig. (1-sided) |
| Pearson Chi-Square | 19.933^a^ | 1 | .000 |  |  |
| Continuity Correction^b^ | 17.590 | 1 | .000 |  |  |
| Likelihood Ratio | 22.048 | 1 | .000 |  |  |
| Fisher's Exact Test |  |  |  | .000 | .000 |
| Linear-by-Linear Association | 19.777 | 1 | .000 |  |  |
| N of Valid Cases | 128 |  |  |  |  |
| a. 0 cells (0.0%) have expected count less than 5. The minimum expected count is 6.75. | | | | | |
| b. Computed only for a 2x2 table | | | | | |

**chauffeur driven only * Melbourne Area Recoded**

| **Crosstab** | | | | | |
| --- | --- | --- | --- | --- | --- |
|  | | | Melbourne Area Recoded | | Total |
|  |  |  | Yes | No |  |
| chauffeur driven only | yes | Count | 10 | 64 | 74 |
|  |  | Expected Count | 12.7 | 61.3 | 74.0 |
|  | no | Count | 12 | 42 | 54 |
|  |  | Expected Count | 9.3 | 44.7 | 54.0 |
| Total | | Count | 22 | 106 | 128 |
|  |  | Expected Count | 22.0 | 106.0 | 128.0 |

| **Chi-Square Tests** | | | | | |
| --- | --- | --- | --- | --- | --- |
|  | Value | df | Asymptotic Significance (2-sided) | Exact Sig. (2-sided) | Exact Sig. (1-sided) |
| Pearson Chi-Square | 1.663^a^ | 1 | .197 |  |  |
| Continuity Correction^b^ | 1.108 | 1 | .293 |  |  |
| Likelihood Ratio | 1.644 | 1 | .200 |  |  |
| Fisher's Exact Test |  |  |  | .238 | .146 |
| Linear-by-Linear Association | 1.650 | 1 | .199 |  |  |
| N of Valid Cases | 128 |  |  |  |  |
| a. 0 cells (0.0%) have expected count less than 5. The minimum expected count is 9.28. | | | | | |
| b. Computed only for a 2x2 table | | | | | |

**chauffeur driven only * Sydney Recoded**

| **Crosstab** | | | | | |
| --- | --- | --- | --- | --- | --- |
|  | | | Sydney Recoded | | Total |
|  |  |  | Yes | No |  |
| chauffeur driven only | yes | Count | 15 | 59 | 74 |
|  |  | Expected Count | 8.7 | 65.3 | 74.0 |
|  | no | Count | 0 | 54 | 54 |
|  |  | Expected Count | 6.3 | 47.7 | 54.0 |
| Total | | Count | 15 | 113 | 128 |
|  |  | Expected Count | 15.0 | 113.0 | 128.0 |

| **Chi-Square Tests** | | | | | |
| --- | --- | --- | --- | --- | --- |
|  | Value | df | Asymptotic Significance (2-sided) | Exact Sig. (2-sided) | Exact Sig. (1-sided) |
| Pearson Chi-Square | 12.399^a^ | 1 | .000 |  |  |
| Continuity Correction^b^ | 10.517 | 1 | .001 |  |  |
| Likelihood Ratio | 17.878 | 1 | .000 |  |  |
| Fisher's Exact Test |  |  |  | .000 | .000 |
| Linear-by-Linear Association | 12.302 | 1 | .000 |  |  |
| N of Valid Cases | 128 |  |  |  |  |
| a. 0 cells (0.0%) have expected count less than 5. The minimum expected count is 6.33. | | | | | |
| b. Computed only for a 2x2 table | | | | | |

…………………………………………….

1. **CHI SQAURED ALL DRIVING PATTERNS**

……………………………………………………..

GET

FILE='C:\Users\ifedi\OneDrive\Documents\Researches\Results and SPSS Outputs\Doctor Risk Results Data SPSS MAIN.sav'.

DATASET NAME DataSet1 WINDOW=FRONT.

CROSSTABS

/TABLES=driveselfYorN chauffeurdriveYorN driveselfandchaperone BY AdelaideR BrisbaneAreaR

GoldCoastR MelbourneAreaR SydneyR

/FORMAT=AVALUE TABLES

/STATISTICS=CHISQ

/CELLS=COUNT

/COUNT ROUND CELL.

**Crosstabs**

| **Notes** | | |
| --- | --- | --- |
| Output Created | | 13-FEB-2017 12:21:58 |
| Comments | |  |
| Input | Data | C:\Users\ifedi\OneDrive\Documents\Researches\Results and SPSS Outputs\Doctor Risk Results Data SPSS MAIN.sav |
|  | Active Dataset | DataSet1 |
|  | Filter | <none> |
|  | Weight | <none> |
|  | Split File | <none> |
|  | N of Rows in Working Data File | 172 |
| Missing Value Handling | Definition of Missing | User-defined missing values are treated as missing. |
|  | Cases Used | Statistics for each table are based on all the cases with valid data in the specified range(s) for all variables in each table. |
| Syntax | | CROSSTABS  /TABLES=driveselfYorN chauffeurdriveYorN driveselfandchaperone BY AdelaideR BrisbaneAreaR  GoldCoastR MelbourneAreaR SydneyR  /FORMAT=AVALUE TABLES  /STATISTICS=CHISQ  /CELLS=COUNT  /COUNT ROUND CELL. |
| Resources | Processor Time | 00:00:00.02 |
|  | Elapsed Time | 00:00:00.16 |
|  | Dimensions Requested | 2 |
|  | Cells Available | 524245 |

[DataSet1] C:\Users\ifedi\OneDrive\Documents\Researches\Results and SPSS Outputs\Doctor Risk Results Data SPSS MAIN.sav

| **Case Processing Summary** | | | | | | |
| --- | --- | --- | --- | --- | --- | --- |
|  | Cases | | | | | |
|  | Valid | | Missing | | Total | |
|  | N | Percent | N | Percent | N | Percent |
| Drive Self Yes or No * Adelaide recoded | 125 | 72.7% | 47 | 27.3% | 172 | 100.0% |
| Drive Self Yes or No * Brisbane Area Recoded | 125 | 72.7% | 47 | 27.3% | 172 | 100.0% |
| Drive Self Yes or No * Gold Coast recoded | 125 | 72.7% | 47 | 27.3% | 172 | 100.0% |
| Drive Self Yes or No * Melbourne Area Recoded | 125 | 72.7% | 47 | 27.3% | 172 | 100.0% |
| Drive Self Yes or No * Sydney Recoded | 125 | 72.7% | 47 | 27.3% | 172 | 100.0% |
| chauffeur driven only * Adelaide recoded | 128 | 74.4% | 44 | 25.6% | 172 | 100.0% |
| chauffeur driven only * Brisbane Area Recoded | 128 | 74.4% | 44 | 25.6% | 172 | 100.0% |
| chauffeur driven only * Gold Coast recoded | 128 | 74.4% | 44 | 25.6% | 172 | 100.0% |
| chauffeur driven only * Melbourne Area Recoded | 128 | 74.4% | 44 | 25.6% | 172 | 100.0% |
| chauffeur driven only * Sydney Recoded | 128 | 74.4% | 44 | 25.6% | 172 | 100.0% |
| Self and Chaperone Driven * Adelaide recoded | 86 | 50.0% | 86 | 50.0% | 172 | 100.0% |
| Self and Chaperone Driven * Brisbane Area Recoded | 86 | 50.0% | 86 | 50.0% | 172 | 100.0% |
| Self and Chaperone Driven * Gold Coast recoded | 86 | 50.0% | 86 | 50.0% | 172 | 100.0% |
| Self and Chaperone Driven * Melbourne Area Recoded | 86 | 50.0% | 86 | 50.0% | 172 | 100.0% |
| Self and Chaperone Driven * Sydney Recoded | 86 | 50.0% | 86 | 50.0% | 172 | 100.0% |

**Drive Self Yes or No * Adelaide recoded**

| **Crosstab** | | | | |
| --- | --- | --- | --- | --- |
| Count | | | | |
|  | | Adelaide recoded | | Total |
|  |  | Yes | No |  |
| Drive Self Yes or No | yes | 29 | 39 | 68 |
|  | no | 12 | 45 | 57 |
| Total | | 41 | 84 | 125 |

| **Chi-Square Tests** | | | | | |
| --- | --- | --- | --- | --- | --- |
|  | Value | df | Asymptotic Significance (2-sided) | Exact Sig. (2-sided) | Exact Sig. (1-sided) |
| Pearson Chi-Square | 6.560^a^ | 1 | .010 |  |  |
| Continuity Correction^b^ | 5.617 | 1 | .018 |  |  |
| Likelihood Ratio | 6.726 | 1 | .010 |  |  |
| Fisher's Exact Test |  |  |  | .013 | .008 |
| Linear-by-Linear Association | 6.508 | 1 | .011 |  |  |
| N of Valid Cases | 125 |  |  |  |  |
| a. 0 cells (0.0%) have expected count less than 5. The minimum expected count is 18.70. | | | | | |
| b. Computed only for a 2x2 table | | | | | |

**Drive Self Yes or No * Brisbane Area Recoded**

| **Crosstab** | | | | |
| --- | --- | --- | --- | --- |
| Count | | | | |
|  | | Brisbane Area Recoded | | Total |
|  |  | Yes | No |  |
| Drive Self Yes or No | yes | 2 | 66 | 68 |
|  | no | 23 | 34 | 57 |
| Total | | 25 | 100 | 125 |

| **Chi-Square Tests** | | | | | |
| --- | --- | --- | --- | --- | --- |
|  | Value | df | Asymptotic Significance (2-sided) | Exact Sig. (2-sided) | Exact Sig. (1-sided) |
| Pearson Chi-Square | 27.122^a^ | 1 | .000 |  |  |
| Continuity Correction^b^ | 24.834 | 1 | .000 |  |  |
| Likelihood Ratio | 30.172 | 1 | .000 |  |  |
| Fisher's Exact Test |  |  |  | .000 | .000 |
| Linear-by-Linear Association | 26.905 | 1 | .000 |  |  |
| N of Valid Cases | 125 |  |  |  |  |
| a. 0 cells (0.0%) have expected count less than 5. The minimum expected count is 11.40. | | | | | |
| b. Computed only for a 2x2 table | | | | | |

**Drive Self Yes or No * Gold Coast recoded**

| **Crosstab** | | | | |
| --- | --- | --- | --- | --- |
| Count | | | | |
|  | | Gold Coast recoded | | Total |
|  |  | Yes | No |  |
| Drive Self Yes or No | yes | 21 | 47 | 68 |
|  | no | 2 | 55 | 57 |
| Total | | 23 | 102 | 125 |

| **Chi-Square Tests** | | | | | |
| --- | --- | --- | --- | --- | --- |
|  | Value | df | Asymptotic Significance (2-sided) | Exact Sig. (2-sided) | Exact Sig. (1-sided) |
| Pearson Chi-Square | 15.475^a^ | 1 | .000 |  |  |
| Continuity Correction^b^ | 13.705 | 1 | .000 |  |  |
| Likelihood Ratio | 17.953 | 1 | .000 |  |  |
| Fisher's Exact Test |  |  |  | .000 | .000 |
| Linear-by-Linear Association | 15.351 | 1 | .000 |  |  |
| N of Valid Cases | 125 |  |  |  |  |
| a. 0 cells (0.0%) have expected count less than 5. The minimum expected count is 10.49. | | | | | |
| b. Computed only for a 2x2 table | | | | | |

**Drive Self Yes or No * Melbourne Area Recoded**

| **Crosstab** | | | | |
| --- | --- | --- | --- | --- |
| Count | | | | |
|  | | Melbourne Area Recoded | | Total |
|  |  | Yes | No |  |
| Drive Self Yes or No | yes | 15 | 53 | 68 |
|  | no | 13 | 44 | 57 |
| Total | | 28 | 97 | 125 |

| **Chi-Square Tests** | | | | | |
| --- | --- | --- | --- | --- | --- |
|  | Value | df | Asymptotic Significance (2-sided) | Exact Sig. (2-sided) | Exact Sig. (1-sided) |
| Pearson Chi-Square | .010^a^ | 1 | .920 |  |  |
| Continuity Correction^b^ | .000 | 1 | 1.000 |  |  |
| Likelihood Ratio | .010 | 1 | .920 |  |  |
| Fisher's Exact Test |  |  |  | 1.000 | .544 |
| Linear-by-Linear Association | .010 | 1 | .921 |  |  |
| N of Valid Cases | 125 |  |  |  |  |
| a. 0 cells (0.0%) have expected count less than 5. The minimum expected count is 12.77. | | | | | |
| b. Computed only for a 2x2 table | | | | | |

**Drive Self Yes or No * Sydney Recoded**

| **Crosstab** | | | | |
| --- | --- | --- | --- | --- |
| Count | | | | |
|  | | Sydney Recoded | | Total |
|  |  | Yes | No |  |
| Drive Self Yes or No | yes | 1 | 67 | 68 |
|  | no | 7 | 50 | 57 |
| Total | | 8 | 117 | 125 |

| **Chi-Square Tests** | | | | | |
| --- | --- | --- | --- | --- | --- |
|  | Value | df | Asymptotic Significance (2-sided) | Exact Sig. (2-sided) | Exact Sig. (1-sided) |
| Pearson Chi-Square | 6.049^a^ | 1 | .014 |  |  |
| Continuity Correction^b^ | 4.379 | 1 | .036 |  |  |
| Likelihood Ratio | 6.572 | 1 | .010 |  |  |
| Fisher's Exact Test |  |  |  | .023 | .017 |
| Linear-by-Linear Association | 6.001 | 1 | .014 |  |  |
| N of Valid Cases | 125 |  |  |  |  |
| a. 2 cells (50.0%) have expected count less than 5. The minimum expected count is 3.65. | | | | | |
| b. Computed only for a 2x2 table | | | | | |

**chauffeur driven only * Adelaide recoded**

| **Crosstab** | | | | |
| --- | --- | --- | --- | --- |
| Count | | | | |
|  | | Adelaide recoded | | Total |
|  |  | Yes | No |  |
| chauffeur driven only | yes | 17 | 57 | 74 |
|  | no | 23 | 31 | 54 |
| Total | | 40 | 88 | 128 |

| **Chi-Square Tests** | | | | | |
| --- | --- | --- | --- | --- | --- |
|  | Value | df | Asymptotic Significance (2-sided) | Exact Sig. (2-sided) | Exact Sig. (1-sided) |
| Pearson Chi-Square | 5.593^a^ | 1 | .018 |  |  |
| Continuity Correction^b^ | 4.717 | 1 | .030 |  |  |
| Likelihood Ratio | 5.563 | 1 | .018 |  |  |
| Fisher's Exact Test |  |  |  | .021 | .015 |
| Linear-by-Linear Association | 5.550 | 1 | .018 |  |  |
| N of Valid Cases | 128 |  |  |  |  |
| a. 0 cells (0.0%) have expected count less than 5. The minimum expected count is 16.88. | | | | | |
| b. Computed only for a 2x2 table | | | | | |

**chauffeur driven only * Brisbane Area Recoded**

| **Crosstab** | | | | |
| --- | --- | --- | --- | --- |
| Count | | | | |
|  | | Brisbane Area Recoded | | Total |
|  |  | Yes | No |  |
| chauffeur driven only | yes | 31 | 43 | 74 |
|  | no | 4 | 50 | 54 |
| Total | | 35 | 93 | 128 |

| **Chi-Square Tests** | | | | | |
| --- | --- | --- | --- | --- | --- |
|  | Value | df | Asymptotic Significance (2-sided) | Exact Sig. (2-sided) | Exact Sig. (1-sided) |
| Pearson Chi-Square | 18.687^a^ | 1 | .000 |  |  |
| Continuity Correction^b^ | 16.991 | 1 | .000 |  |  |
| Likelihood Ratio | 21.033 | 1 | .000 |  |  |
| Fisher's Exact Test |  |  |  | .000 | .000 |
| Linear-by-Linear Association | 18.541 | 1 | .000 |  |  |
| N of Valid Cases | 128 |  |  |  |  |
| a. 0 cells (0.0%) have expected count less than 5. The minimum expected count is 14.77. | | | | | |
| b. Computed only for a 2x2 table | | | | | |

**chauffeur driven only * Gold Coast recoded**

| **Crosstab** | | | | |
| --- | --- | --- | --- | --- |
| Count | | | | |
|  | | Gold Coast recoded | | Total |
|  |  | Yes | No |  |
| chauffeur driven only | yes | 1 | 73 | 74 |
|  | no | 15 | 39 | 54 |
| Total | | 16 | 112 | 128 |

| **Chi-Square Tests** | | | | | |
| --- | --- | --- | --- | --- | --- |
|  | Value | df | Asymptotic Significance (2-sided) | Exact Sig. (2-sided) | Exact Sig. (1-sided) |
| Pearson Chi-Square | 19.933^a^ | 1 | .000 |  |  |
| Continuity Correction^b^ | 17.590 | 1 | .000 |  |  |
| Likelihood Ratio | 22.048 | 1 | .000 |  |  |
| Fisher's Exact Test |  |  |  | .000 | .000 |
| Linear-by-Linear Association | 19.777 | 1 | .000 |  |  |
| N of Valid Cases | 128 |  |  |  |  |
| a. 0 cells (0.0%) have expected count less than 5. The minimum expected count is 6.75. | | | | | |
| b. Computed only for a 2x2 table | | | | | |

**chauffeur driven only * Melbourne Area Recoded**

| **Crosstab** | | | | |
| --- | --- | --- | --- | --- |
| Count | | | | |
|  | | Melbourne Area Recoded | | Total |
|  |  | Yes | No |  |
| chauffeur driven only | yes | 10 | 64 | 74 |
|  | no | 12 | 42 | 54 |
| Total | | 22 | 106 | 128 |

| **Chi-Square Tests** | | | | | |
| --- | --- | --- | --- | --- | --- |
|  | Value | df | Asymptotic Significance (2-sided) | Exact Sig. (2-sided) | Exact Sig. (1-sided) |
| Pearson Chi-Square | 1.663^a^ | 1 | .197 |  |  |
| Continuity Correction^b^ | 1.108 | 1 | .293 |  |  |
| Likelihood Ratio | 1.644 | 1 | .200 |  |  |
| Fisher's Exact Test |  |  |  | .238 | .146 |
| Linear-by-Linear Association | 1.650 | 1 | .199 |  |  |
| N of Valid Cases | 128 |  |  |  |  |
| a. 0 cells (0.0%) have expected count less than 5. The minimum expected count is 9.28. | | | | | |
| b. Computed only for a 2x2 table | | | | | |

**chauffeur driven only * Sydney Recoded**

| **Crosstab** | | | | |
| --- | --- | --- | --- | --- |
| Count | | | | |
|  | | Sydney Recoded | | Total |
|  |  | Yes | No |  |
| chauffeur driven only | yes | 15 | 59 | 74 |
|  | no | 0 | 54 | 54 |
| Total | | 15 | 113 | 128 |

| **Chi-Square Tests** | | | | | |
| --- | --- | --- | --- | --- | --- |
|  | Value | df | Asymptotic Significance (2-sided) | Exact Sig. (2-sided) | Exact Sig. (1-sided) |
| Pearson Chi-Square | 12.399^a^ | 1 | .000 |  |  |
| Continuity Correction^b^ | 10.517 | 1 | .001 |  |  |
| Likelihood Ratio | 17.878 | 1 | .000 |  |  |
| Fisher's Exact Test |  |  |  | .000 | .000 |
| Linear-by-Linear Association | 12.302 | 1 | .000 |  |  |
| N of Valid Cases | 128 |  |  |  |  |
| a. 0 cells (0.0%) have expected count less than 5. The minimum expected count is 6.33. | | | | | |
| b. Computed only for a 2x2 table | | | | | |

**Self and Chaperone Driven * Adelaide recoded**

| **Crosstab** | | | | |
| --- | --- | --- | --- | --- |
| Count | | | | |
|  | | Adelaide recoded | | Total |
|  |  | Yes | No |  |
| Self and Chaperone Driven | yes | 4 | 3 | 7 |
|  | no | 30 | 49 | 79 |
| Total | | 34 | 52 | 86 |

| **Chi-Square Tests** | | | | | |
| --- | --- | --- | --- | --- | --- |
|  | Value | df | Asymptotic Significance (2-sided) | Exact Sig. (2-sided) | Exact Sig. (1-sided) |
| Pearson Chi-Square | .988^a^ | 1 | .320 |  |  |
| Continuity Correction^b^ | .349 | 1 | .555 |  |  |
| Likelihood Ratio | .963 | 1 | .327 |  |  |
| Fisher's Exact Test |  |  |  | .427 | .273 |
| Linear-by-Linear Association | .977 | 1 | .323 |  |  |
| N of Valid Cases | 86 |  |  |  |  |
| a. 2 cells (50.0%) have expected count less than 5. The minimum expected count is 2.77. | | | | | |
| b. Computed only for a 2x2 table | | | | | |

**Self and Chaperone Driven * Brisbane Area Recoded**

| **Crosstab** | | | | |
| --- | --- | --- | --- | --- |
| Count | | | | |
|  | | Brisbane Area Recoded | | Total |
|  |  | Yes | No |  |
| Self and Chaperone Driven | yes | 2 | 5 | 7 |
|  | no | 16 | 63 | 79 |
| Total | | 18 | 68 | 86 |

| **Chi-Square Tests** | | | | | |
| --- | --- | --- | --- | --- | --- |
|  | Value | df | Asymptotic Significance (2-sided) | Exact Sig. (2-sided) | Exact Sig. (1-sided) |
| Pearson Chi-Square | .269^a^ | 1 | .604 |  |  |
| Continuity Correction^b^ | .001 | 1 | .973 |  |  |
| Likelihood Ratio | .251 | 1 | .617 |  |  |
| Fisher's Exact Test |  |  |  | .633 | .453 |
| Linear-by-Linear Association | .266 | 1 | .606 |  |  |
| N of Valid Cases | 86 |  |  |  |  |
| a. 1 cells (25.0%) have expected count less than 5. The minimum expected count is 1.47. | | | | | |
| b. Computed only for a 2x2 table | | | | | |

**Self and Chaperone Driven * Gold Coast recoded**

| **Crosstab** | | | | |
| --- | --- | --- | --- | --- |
| Count | | | | |
|  | | Gold Coast recoded | | Total |
|  |  | Yes | No |  |
| Self and Chaperone Driven | yes | 0 | 7 | 7 |
|  | no | 11 | 68 | 79 |
| Total | | 11 | 75 | 86 |

| **Chi-Square Tests** | | | | | |
| --- | --- | --- | --- | --- | --- |
|  | Value | df | Asymptotic Significance (2-sided) | Exact Sig. (2-sided) | Exact Sig. (1-sided) |
| Pearson Chi-Square | 1.118^a^ | 1 | .290 |  |  |
| Continuity Correction^b^ | .218 | 1 | .641 |  |  |
| Likelihood Ratio | 2.005 | 1 | .157 |  |  |
| Fisher's Exact Test |  |  |  | .588 | .369 |
| Linear-by-Linear Association | 1.105 | 1 | .293 |  |  |
| N of Valid Cases | 86 |  |  |  |  |
| a. 1 cells (25.0%) have expected count less than 5. The minimum expected count is .90. | | | | | |
| b. Computed only for a 2x2 table | | | | | |

**Self and Chaperone Driven * Melbourne Area Recoded**

| **Crosstab** | | | | |
| --- | --- | --- | --- | --- |
| Count | | | | |
|  | | Melbourne Area Recoded | | Total |
|  |  | Yes | No |  |
| Self and Chaperone Driven | yes | 0 | 7 | 7 |
|  | no | 18 | 61 | 79 |
| Total | | 18 | 68 | 86 |

| **Chi-Square Tests** | | | | | |
| --- | --- | --- | --- | --- | --- |
|  | Value | df | Asymptotic Significance (2-sided) | Exact Sig. (2-sided) | Exact Sig. (1-sided) |
| Pearson Chi-Square | 2.017^a^ | 1 | .156 |  |  |
| Continuity Correction^b^ | .875 | 1 | .349 |  |  |
| Likelihood Ratio | 3.449 | 1 | .063 |  |  |
| Fisher's Exact Test |  |  |  | .337 | .180 |
| Linear-by-Linear Association | 1.994 | 1 | .158 |  |  |
| N of Valid Cases | 86 |  |  |  |  |
| a. 1 cells (25.0%) have expected count less than 5. The minimum expected count is 1.47. | | | | | |
| b. Computed only for a 2x2 table | | | | | |

**Self and Chaperone Driven * Sydney Recoded**

| **Crosstab** | | | | |
| --- | --- | --- | --- | --- |
| Count | | | | |
|  | | Sydney Recoded | | Total |
|  |  | Yes | No |  |
| Self and Chaperone Driven | yes | 1 | 6 | 7 |
|  | no | 4 | 75 | 79 |
| Total | | 5 | 81 | 86 |

| **Chi-Square Tests** | | | | | |
| --- | --- | --- | --- | --- | --- |
|  | Value | df | Asymptotic Significance (2-sided) | Exact Sig. (2-sided) | Exact Sig. (1-sided) |
| Pearson Chi-Square | .999^a^ | 1 | .318 |  |  |
| Continuity Correction^b^ | .025 | 1 | .875 |  |  |
| Likelihood Ratio | .752 | 1 | .386 |  |  |
| Fisher's Exact Test |  |  |  | .353 | .353 |
| Linear-by-Linear Association | .987 | 1 | .320 |  |  |
| N of Valid Cases | 86 |  |  |  |  |
| a. 2 cells (50.0%) have expected count less than 5. The minimum expected count is .41. | | | | | |
| b. Computed only for a 2x2 table | | | | | |

………………………………………………………………………………..

1. **ASSOCIATIONS: MIXED**

| **Variables in the Equation** | | | | | | | | | |
| --- | --- | --- | --- | --- | --- | --- | --- | --- | --- |
|  | | B | S.E. | Wald | df | Sig. | Exp(B) | 95% C.I.for EXP(B) | |
|  |  |  |  |  |  |  |  | Lower | Upper |
| Step 1^a^ | Concerned recoded 2 | .551 | .609 | .818 | 1 | .366 | 1.735 | .526 | 5.727 |
|  | Apprehension Recoded 2 | .820 | .575 | 2.032 | 1 | .154 | 2.270 | .735 | 7.008 |
|  | Constant | -2.876 | .956 | 9.052 | 1 | .003 | .056 |  |  |
| a. Variable(s) entered on step 1: Concerned recoded 2, Apprehension Recoded 2. | | | | | | | | | |

| **Variables in the Equation** | | | | | | | | | | | | | | | | | |  |
| --- | --- | --- | --- | --- | --- | --- | --- | --- | --- | --- | --- | --- | --- | --- | --- | --- | --- | --- |
|  | | B | | S.E. | | Wald | | df | | Sig. | | Exp(B) | | 95% C.I.for EXP(B) | | | |  |
|  |  |  |  |  |  |  |  |  |  |  |  |  |  | Lower | | Upper | |  |
| Step 1^a^ | Concerned recoded 2 | .999 | | .514 | | 3.770 | | 1 | | .052 | | 2.714 | | .991 | | 7.437 | |  |
|  | Constant | -2.420 | | .892 | | 7.352 | | 1 | | .007 | | .089 | |  | |  | |  |
| a. Variable(s) entered on step 1: Concerned recoded 2. | | | | | | | | | | | | | | | | | |  |
| **Variables in the Equation** | | | | | | | | | | | | | | | | | | |
|  | | | B | | S.E. | | Wald | | df | | Sig. | | Exp(B) | | 95% C.I.for EXP(B) | | | |
|  |  |  |  |  |  |  |  |  |  |  |  |  |  |  | Lower | | Upper | |
| Step 1^a^ | Apprehension Recoded 2 | | 1.109 | | .488 | | 5.162 | | 1 | | .023 | | 3.030 | | 1.164 | | 7.886 | |
|  | Constant | | -2.400 | | .765 | | 9.829 | | 1 | | .002 | | .091 | |  | |  | |
| a. Variable(s) entered on step 1: Apprehension Recoded 2. | | | | | | | | | | | | | | | | | | |

………………………………………………………………………………………………………

1. **ASSOCIATIONS: CHAUFFEUR ONLY**

| **Variables in the Equation** | | | | | | | | | |
| --- | --- | --- | --- | --- | --- | --- | --- | --- | --- |
|  | | B | S.E. | Wald | df | Sig. | Exp(B) | 95% C.I.for EXP(B) | |
|  |  |  |  |  |  |  |  | Lower | Upper |
| Step 1^a^ | Apprehension Recoded 2 | 1.163 | .476 | 5.960 | 1 | .015 | 3.199 | 1.258 | 8.139 |
|  | Constant | -.511 | .640 | .637 | 1 | .425 | .600 |  |  |
| a. Variable(s) entered on step 1: Apprehension Recoded 2. | | | | | | | | | |

| **Variables in the Equation** | | | | | | | | | |
| --- | --- | --- | --- | --- | --- | --- | --- | --- | --- |
|  | | B | S.E. | Wald | df | Sig. | Exp(B) | 95% C.I.for EXP(B) | |
|  |  |  |  |  |  |  |  | Lower | Upper |
| Step 1^a^ | degreeR | .919 | .420 | 4.781 | 1 | .029 | 2.506 | 1.100 | 5.708 |
|  | Constant | -.491 | .712 | .476 | 1 | .490 | .612 |  |  |
| a. Variable(s) entered on step 1: degreeR. | | | | | | | | | |

| **Variables in the Equation** | | | | | | | | | |
| --- | --- | --- | --- | --- | --- | --- | --- | --- | --- |
|  | | B | S.E. | Wald | df | Sig. | Exp(B) | 95% C.I.for EXP(B) | |
|  |  |  |  |  |  |  |  | Lower | Upper |
| Step 1^a^ | durationR | -1.074 | .421 | 6.509 | 1 | .011 | .342 | .150 | .780 |
|  | Constant | 2.702 | .710 | 14.494 | 1 | .000 | 14.902 |  |  |
| a. Variable(s) entered on step 1: durationR. | | | | | | | | | |

| **Variables in the Equation** | | | | | | | | | |
| --- | --- | --- | --- | --- | --- | --- | --- | --- | --- |
|  | | B | S.E. | Wald | df | Sig. | Exp(B) | 95% C.I.for EXP(B) | |
|  |  |  |  |  |  |  |  | Lower | Upper |
| Step 1^a^ | Registration Status | 1.233 | .449 | 7.545 | 1 | .006 | 3.430 | 1.423 | 8.265 |
|  | Constant | -.876 | .666 | 1.727 | 1 | .189 | .416 |  |  |
| a. Variable(s) entered on step 1: Registration Status. | | | | | | | | | |

| **Variables in the Equation** | | | | | | | | | |
| --- | --- | --- | --- | --- | --- | --- | --- | --- | --- |
|  | | B | S.E. | Wald | df | Sig. | Exp(B) | 95% C.I.for EXP(B) | |
|  |  |  |  |  |  |  |  | Lower | Upper |
| Step 1^a^ | Gender | 1.674 | .766 | 4.775 | 1 | .029 | 5.333 | 1.188 | 23.936 |
|  | Constant | -.863 | .850 | 1.031 | 1 | .310 | .422 |  |  |
| a. Variable(s) entered on step 1: Gender. | | | | | | | | | |

| **Variables in the Equation** | | | | | | | | | |
| --- | --- | --- | --- | --- | --- | --- | --- | --- | --- |
|  | | B | S.E. | Wald | df | Sig. | Exp(B) | 95% C.I.for EXP(B) | |
|  |  |  |  |  |  |  |  | Lower | Upper |
| Step 1^a^ | Gender | 1.770 | .828 | 4.570 | 1 | .033 | 5.872 | 1.159 | 29.767 |
|  | Registration Status | .578 | .618 | .873 | 1 | .350 | 1.782 | .531 | 5.983 |
|  | durationR | -.662 | .600 | 1.215 | 1 | .270 | .516 | .159 | 1.673 |
|  | degreeR | .690 | .554 | 1.552 | 1 | .213 | 1.994 | .673 | 5.905 |
|  | apprehensionR | 1.132 | .552 | 4.201 | 1 | .040 | 3.101 | 1.051 | 9.153 |
|  | Constant | -3.971 | 2.085 | 3.627 | 1 | .057 | .019 |  |  |
| a. Variable(s) entered on step 1: Gender, Registration Status, durationR, degreeR, apprehensionR. | | | | | | | | | |

…………………………………………………………………………………………………………………………….

1. **ASSOCIATIONS: SELF-DRIVE ONLY**

| **Variables in the Equation** | | | | | | | | | | | | | | | | | | | | | | | | | | | |  |
| --- | --- | --- | --- | --- | --- | --- | --- | --- | --- | --- | --- | --- | --- | --- | --- | --- | --- | --- | --- | --- | --- | --- | --- | --- | --- | --- | --- | --- |
|  | | | B | | | S.E. | | | Wald | | | df | | | Sig. | | | | Exp(B) | | | 95% C.I.for EXP(B) | | | | | |  |
|  |  |  |  |  |  |  |  |  |  |  |  |  |  |  |  |  |  |  |  |  |  | Lower | | | Upper | | |  |
| Step 1^a^ | | Gender | -1.757 | | | .515 | | | 11.626 | | | 1 | | | .001 | | | | .173 | | | .063 | | | .474 | | |  |
|  |  | Constant | 3.109 | | | .661 | | | 22.104 | | | 1 | | | .000 | | | | 22.392 | | |  | | |  | | |  |
| a. Variable(s) entered on step 1: Gender. | | | | | | | | | | | | | | | | | | | | | | | | | | | |  |
| **Variables in the Equation** | | | | | | | | | | | | | | | | | | | | | | | | | | | | |
|  | | | | | B | | | S.E. | | | Wald | | | df | | | Sig. | | | Exp(B) | | | | 95% C.I.for EXP(B) | | | | |
|  |  |  |  |  |  |  |  |  |  |  |  |  |  |  |  |  |  |  |  |  |  |  |  | Lower | | | Upper | |
| Step 1^a^ | | age range recoded | | | -.897 | | | .430 | | | 4.356 | | | 1 | | | .037 | | | .408 | | | | .176 | | | .947 | |
|  |  | Constant | | | 2.445 | | | .741 | | | 10.894 | | | 1 | | | .001 | | | 11.525 | | | |  | | |  | |
| 1. Variable(s) entered on step 1: age range recoded. | | | | | | | | | | | | | | | | | | | | | | | | | | | | |
| **Variables in the Equation**  **Male Vs Female** | | | | | | | | | | | | | | | | | | | | | | | | | |  |  |  |
|  | | | | B | | | S.E. | | | Wald | | | df | | | Sig. | | Exp(B) | | | 95% C.I.for EXP(B) | | | | |  |  |  |
|  |  |  |  |  |  |  |  |  |  |  |  |  |  |  |  |  |  |  |  |  | Lower | | Upper | | |  |  |  |
| Step 1^a^ | Gender | | | -1.601 | | | .527 | | | 9.240 | | | 1 | | | .002 | | .202 | | | .072 | | .566 | | |  |  |  |
|  | age range recoded | | | -.651 | | | .451 | | | 2.078 | | | 1 | | | .149 | | .522 | | | .215 | | 1.264 | | |  |  |  |
|  | Constant | | | 3.962 | | | .922 | | | 18.458 | | | 1 | | | .000 | | 52.589 | | |  | |  | | |  |  |  |
| a. Variable(s) entered on step 1: Gender, age range recoded. | | | | | | | | | | | | | | | | | | | | | | | | | |  |  |  |
