# Supplementary material for: The determinants and engagement patterns of chaperones and chauffeurs by Australian doctors in after-hours house-call services
Source: PeerJ. 2017 Apr 26;5:e3218. doi: 10.7717/peerj.3218 (PMC5408717; doi:10.7717/peerj.3218)
Supplement: Data S1 [file peerj-05-3218-s002.pdf]

Doctors' Safety, Satisfaction and Stress in after-hour house call services

1. Welcome to My Survey. Introduction and Consent

Dear,

Thank you very much for agreeing to participate in this survey.

This is a study being conducted by Dr. Chris Ifediora, a General Practitioner and a Senior Lecturer at the Griffith University Medical School.

The study aims to assess the perception and safety of doctors undertaking after-hours house calls, as well as their satisfaction and stress levels. The sample size includes all doctors registered with, and working for the National Home Doctor Service.The researcher himself also takes part in after-hours house calls.

The survey is expected to take about 10 minutes to complete, and apart from this time consideration, no risks or discomforts are anticipated from taking part.

It is hoped that the findings will help proffer suggestions and influence key policies on after-hour house call services, and will help interested doctor's wishing to undertake such services in the future make informed decisions.

A copy of the finished work will be made available to all interested Participants through the National Home Doctors Service, and will be emailed to you through the same email address available to the company.

Your participation in this study is voluntary, and strict confidentiality will be maintained throughout.

Ethical clearance has been obtained from the Griffith University Human Research Ethics Committee, with reference numbers MED/34/14/HREC and MED/47/14/HREC.

The collated, anonymized data and computed results will be stored in the Griffith University Research Storage Service in the active phase of the research. Afterwards, this will be moved to the Griffith Archive Storage system, and will be kept for a duration consistent with the University's regulations.

If you have any concerns with this study, please contact: Dr. Chris O. Ifediora (MBBS, MPH, FRACGP) through "c.ifediora@griffith.edu.au" or 0755808331.
If, however, you have additional questions about the ethics of this research, please contact the Manager, Research Ethics on 3735 4375 or "research-ethics@griffith.edu.au."

Please answer the following question to proceed.

***1. Do you consent to participating in this study?**

2. Biodata

This Section asks questions about your basic information. All information will remain anonymous.

*2. What is your gender?

*3. What is your age?

- <25
- 25 to 39
- 40 to 60
- >60

*4. What best describes your marital status?

- Married
- Single
- Seperated/Divorced
- Co-habiting
- Civil Partnership
- Widowed
- Other (please specify)

5. Which best describes your family setting?

- I have kid(s) and live with them
- I have kid(s) but do not live with them
- I have no kids and/or live with none

3. Professional Data

This Section asks questions about some of your professional background.

*6. What best describes your primary medical qualification?

- Australian Trained
- Overseas: New Zealand
- Overseas: Other

*7. What medical specialty do you primarily belong to?

- General Practitioner (GP)
- Medical Specialty *(Go to Question 9)*
- Surgical Specialty *(Go to Question 9)*
- Emergency Physician *(Go to Question 9)*
- Other *(Go to Question 9)*

Other (please specify)

8. If you are a GP, which best describes your registration status?

- Vocationally Registered (including Fellows of a recognized GP College)
- Non-Vocationally registered (Not a Fellow yet)
- Other

Other (please specify)

*9. Irrespective of your specialty, have you ever undertaken after-hour general practice House Calls anytime in the last twelve months (i.e., from October 1st 2013 to September 30th 2014)? *(After-hours service is defined as medical care provided to patients (i) before 8.00am and after 6pm on weekdays (ii) before 8.00am and after 12.00pm on Saturdays (iii) all day Sundays and public holidays).*

- Yes
- No

***10. Where in Australia did you undertake the majority of your after-hour house calls in the last twelve months?**

- Adelaide
- Brisbane
- Canberra
- Gold Coast
- Geelong
- Melbourne
- Sunshine Coast
- Sydney
- Other

Other (please specify)

11. In the last twelve months, which of the following apply to you when making house calls?** ***Please tick all that apply.

	Not at all	Rarely	Sometimes	Most times	All the time
I drive myself					
I use a Chaffeur					
I drive myself, but usually have a chaperone					

***12. On average in the last twelve months, how many hours of after-hour general practice do you do per week?**

- Less than 24 hours
- 24 to 37.5 hours
- Over 37.5 hours

***13. Altogether, how many years have you been doing after-hour doctor services for?**

- 2 years or less
- Between 2 and 10 years
- Over 10 years

4. Risks

These questions assess your perception of, and actual risks encountered while on after-hour house calls in the past twelve months.

14. With respect to safety, what is your level of concern regarding after-hours house calls?

Not at all concerned	Slightly concerned	Somewhat concerned	Moderately concerned	Extremely concerned
○	○	○	○	○

15. What best describes your disposition when on after-hours duties?

Not at all apprehensive	Slightly apprehensive	Somewhat apprehensive	Moderately apprehensive	Extremely apprehensive
○	○	○	○	○

***16. In the past twelve months, have you faced any kind of aggression while making house calls?**

○ Yes

○ No

○ Not sure

5.

17. If you have answered yes to the above question, what kind of aggression have you experienced from either the patient, his/her family or his/her friends? *Please tick all that apply.*

- [ ] **Verbal Abuse**

(Swearing or using obscene gestures aimed at offending you. This can include abuse over the phone).

- [ ] **Property damage or Theft**

(Damage or theft to property belonging to you, your family or your workplace. Attempted theft is also included).

- [ ] **Physical Violence**

(Being physically attacked. Includes punching, slapping, kicking, or use of a weapon or other object with intent to cause bodily harm or intimidate).

- [ ] **Threats**

(Using words or gestures to threaten, including attempts to pressure you into prescribing or completing a certificate)

- [ ] **Sexual harrassment**

(Any form of sexual proposition or unwelcome sexual attention, including behaviours such as humiliating or offensive jokes and remarks with sexual overtones, suggestive looks or physical gestures, requests for inappropriate physical examinations, and touching or grabbing)

- [ ] **Sexual violence/abuse/assault**

(Any forced sexual act, rape, or indecent assault. Includes brushing, touching, grabbing of the genitals or breasts).

- [ ] **Stalking**

(Being purposefully stalked or followed to or from home or workplace, including to Home Visits and Hospitals).

- [ ] **Vexatious complaints made against you**

(Unnecessary and frivoulous complaints made against you verbally, wriiten or otherwise, whether to your employers or to the Medical Board).

- [ ] Other (please specify)

18. If you have encountered aggression in any way, who was it from? *Please tick all that apply.*

- [ ] The patient him/herself
- [ ] The relatives
- [ ] Friends of the patient

* 19. Do you have any measures in place to protect yourself in case of any problems?

- ○ Yes
- ○ No
- ○ Have never thought about it
- ○ Have thought about it, but unsure of what to do.

20. If you have answered yes to Question 19 above, what kind of self-protective measures do you have in place? ***Please tick all that apply.***

- [ ] Panic Buttons
- [ ] Personal Alarms
- [ ] Use of chaperone/security personnel
- [ ] Had undergone a self-defence or De-escalation Training
- [ ] Dependent on the Surgery/Practice Policies of tracking your movements and/or blocking percieved "risky" patients
- [ ] Other

Other (please specify)

6. Satisfaction

This Section asks questions to assess the level of job satisfaction among after-hour doctors *(Adapted from: 10-item Warr-Cook-Wall questionnaire)*

*21. How satisfied are you with the after-hour house calls with respect to the following? *Please answer all questions.*

	Not at all satisfied	slightly satisfied	moderately satisfied	Very satisfied	Extremely satisfied
Amount of variety involved when on house calls	○	○	○	○	○
Opportunity to use clinical skills	○	○	○	○	○
Freedom of working method and independence	○	○	○	○	○
Amount of responsibility	○	○	○	○	○
Physical working condition	○	○	○	○	○
Hours of work	○	○	○	○	○
Income	○	○	○	○	○
Recognition for work	○	○	○	○	○
Colleagues and fellow workers	○	○	○	○	○
Overall job satisfaction	○	○	○	○	○

*22. Do you feel you have enough clinical and professional support while on the job? *Please answer all questions.*

	No, not at all	Some support	Moderate suppport	Very well supported
Clinical support, including books, information and decision-making resources	○	○	○	○
Professional support, including consulting colleagues and ambulances	○	○	○	○
Security support, including ease of calling for, or getting help if in trouble	○	○	○	○

7. Stress

This Section asks questions to explore the stress levels of doctors undertaking after-hour house calls *(adapted from the Malasch Burnout Inventory, MBI).*

For the next three Questions, please answer: "*Considering your job in after-hour house calls, how often do the following statements describe the way you feel"? (Please answer all)*

*23. Emotional Exhaustion

	Every day	A few times a week	Once a week	A few times a month	Once a month or less	A few times over the past twelve months	Never
I feel emotionally drained from my work	○	○	○	○	○	○	○
I feel used up at the end of the workday	○	○	○	○	○	○	○
I feel fatigued when I get up in the morning and have to face another day on the job	○	○	○	○	○	○	○
Driving round to see patients is really a strain for me	○	○	○	○	○	○	○
I feel burned out from my work	○	○	○	○	○	○	○
I feel frustrated by the job	○	○	○	○	○	○	○
I feel I am working too hard on my job	○	○	○	○	○	○	○
Working with people in this job directly puts too much stress on me	○	○	○	○	○	○	○
I feel like I am at the end of my rope	○	○	○	○	○	○	○

*24. Depersonalization

	Every day	A few times a week	Once a week	A few times a month	Once a month or less	A few times over the past twelve months	Never
I feel I treat some patients as if they were impersonal "objects"	○	○	○	○	○	○	○
I have become more callous toward patients since I took this job	○	○	○	○	○	○	○
I worry that this job is hardening me emotionally	○	○	○	○	○	○	○
I do not really care what happens to some patients	○	○	○	○	○	○	○
I feel patients blame me for some of their problems	○	○	○	○	○	○	○

*25. Personal Accomplishment

	Every day	A few times a week	Once a week	A few times a month	Once a month or less	A few times over the past twelve months	Never
I can easily understand how my patients feel about things	○	○	○	○	○	○	○
I deal very effectively with the problems of my patients	○	○	○	○	○	○	○
I feel I am positively influencing my patients' lives through my work	○	○	○	○	○	○	○
I feel very energetic	○	○	○	○	○	○	○
I can easily create a relaxed atmosphere with my patients	○	○	○	○	○	○	○
I feel exhilarated after working closely with my patients	○	○	○	○	○	○	○
I have accomplished many worthwhile things in this job	○	○	○	○	○	○	○
In my work, I deal with emotional problems very calmly	○	○	○	○	○	○	○

8. Thank You!!!

Thank you for taking out time to complete this Survey. Your gesture is highly appreciated.
